# Physical Activity as a New Tool to Evaluate the Response to Omalizumab and Mepolizumab in Severe Asthmatic Patients: A Pilot Study

**DOI:** 10.3389/fphar.2019.01630

**Published:** 2020-01-24

**Authors:** Giovanna Elisiana Carpagnano, Francesco Sessa, Giulia Scioscia, Donato Lacedonia, Maria Pia Foschino, Maria Pia Venuti, Antonio Ivano Triggiani, Anna Valenzano, Onofrio Resta, Giuseppe Cibelli, Giovanni Messina

**Affiliations:** ^1^ Department of Medical and Surgical Sciences, Institute of Respiratory Diseases, University of Foggia, Foggia, Italy; ^2^ Department of Clinical and Experimental Medicine, University of Foggia, Foggia, Italy; ^3^ Department of Neurosciences, University of Bari, Bari, Italy

**Keywords:** mepolizumab, omalizumab, physical activity, severe asthma, quality of life

## Abstract

Asthma is a chronic inflammatory airway disease, representing one of the most severe pathologies in developed countries. Based on a report of the World Health Organization (WHO), it affects about 300 million people worldwide. Few studies have analyzed the effects of daily life physical activity (PA) levels in patients with asthma: moreover, little research has been carried out on PA levels in patients suffering from severe asthma (SA). This study aimed to investigate the PA levels in two groups of patients suffering from SA; in particular, this study analyzed the changes that occur in patients treated with biologic therapy (BT group) and patients who underwent traditional treatment (TT group) over 6 months. Moreover, this study represents a pilot study because, to the best of our knowledge, it is the first investigation that analyzed if the kind of biologic drug (omalizumab or mepolizumab) can produce differences in the PA levels of SA patients. Fifty SA patients were enrolled and PA parameters were monitored for 6 months. Subjects were divided into two treatment groups: TT (20 patients) and BT (30 patients), the BT group was further subdivided according to the drugs used (15, omalizumab; 15, mepolizumab). During drug treatment, all subjects improved their PA levels: indeed, considering the intragroup variation, the PA levels were significantly higher comparing the T6 levels to baseline (T0, p < 0.01). Considering the intragroup variation, it is very interesting to note that biologic therapy improved PA levels compared to the effects of traditional therapy; while at T0 there were no significant differences in the steps per day (SPD) values between the two groups (T0, p = 0.85), the differences become statistically significant at T1, T3, and T6 (T1, p = 0.019; T3, p = 3.48x10^−6^; T6, p = 4.78x10^−10^). As expected, the same differences were reported analyzing the energy expenditure data. In conclusion, this pilot study reports a positive relationship between biologic drug therapy and PA patterns, even if further studies are needed.

## Introduction

Asthma is a chronic inflammatory airway disease, representing one of the most severe pathologies in developed countries. Based on a report of the World Health Organization (WHO), it affects about 300 million people worldwide (Corren et al., 2019). For a long time, it has been considered as an inflammatory disease and treated with corticosteroids (CSs). The pathological condition of severe asthma (SA) was defined as “the pathological status that requires treatment with a high-dose inhaled CS plus a second controller and/or systemic CS to prevent it from becoming “uncontrolled” or that it remains “uncontrolled’ despite this therapy” (Chung et al., 2014; Monda et al., 2019; Sessa et al., 2019). Following this definition, SA patients are characterized by an inadequate response to standard treatment, increasing health care costs, and morbidity (Wenzel and Busse, 2007). Analyzing the worldwide statistics, following the criterion of no-responders to treatment, about 10% of asthmatics can be classified as severe. For these reasons, several new approaches have been attempted to identify the ideal treatment for SA. With the introduction of biologics, the approach to asthmatic patients has been completely modified, moving researchers toward a new stratified medicine for a homogenous group of patients belonging to a specific pheno-endotype. This new approach, apparently more expensive, reduced patients’ future risk, improving their quality of life (QoL) and socio-economic costs.

Several studies have analyzed the use of drugs targeting immunoglobulin E (IgE) or interleukin (IL)-5 in the treatment of SA. The first monoclonal antibody used in the treatment of severe allergic asthma was omalizumab that showed good results in responders in terms of reduction of exacerbations, improvement of symptoms, lung function, and QoL (Fajt and Wenzel, 2017). In a similar context, IL-5 targeted therapy with mepolizumab, has also been recently proposed for patients belonging to the type 2 endotype, also covering non atopic forms of eosinophilic SA (Pavord et al., 2012).

The principal outcomes used to evaluate the efficacy of biologics are the reduction of exacerbations, the improvement of lung function, reduced symptoms, and QoL improvements.

According to the Global Initiative for Asthma (GINA) recommendations, physical activity (PA) should be considered an important tool in asthma treatment (Reddel et al., 2015). Indeed, PA is usually considered very important in many chronic diseases, reducing morbidity and mortality (Blair et al., 1989; Lee et al., 2012; Messina et al., 2017b; Polito et al., 2018; Sessa et al., 2018b). Several studies have described a positive outcome for those asthma patients who combined biologic therapy with PA: a positive correlation has been described between PA levels and lung function in asthma patients (Ritz et al., 2010; Monda et al., 2017a; Panico et al., 2017; Vanacore et al., 2018).

On the other hand, physical inactivity has been associated with adverse health consequences in asthma patients. Although it is well known that PA has positive effects on lung function and mental health both in healthy subjects and in patients, asthma patients practice sport exercise less than healthy subjects (Avallone and McLeish, 2013).

Few studies have analyzed the effects of daily life PA levels in patients with asthma: moreover, little research has been performed on the PA levels in patients suffering from SA (Cordova-Rivera et al., 2018b; Hennegrave et al., 2018). Generally, it is very important for asthma patients to improve their QoL, especially in cases of SA. To achieve this important objective, both the right biologic therapy and adequate PA should be adopted. As previously described, the use of new therapies, such as biologics, is related to a greater PA level, providing a general improvement in QoL of asthma patients (Hossny et al., 2017).

This study aimed to investigate the PA levels in two groups of patients suffering from SA; particularly, this study analyzed the changes that occur in patients treated with biologic therapy (BT group) and others who underwent traditional treatment (TT group) over 6 months. Moreover, this study represents a pilot study because, to the best of our knowledge, it is the first investigation that analyzed if the kind of biologic drug (omalizumab or mepolizumab) can produce differences in the PA levels of SA patients.

## Materials and Methods

### Study Setting and Participants

This was a cross-sectional study conducted in the accredited outpatient pulmonary clinic for SA of the Institute of Respiratory Diseases of the University of Foggia between December 2016 and March 2019. We enrolled 30 patients (20 females and 10 males, mean age 55.2 ± 11.3 years) with SA diagnosed according to the European Respiratory Society (ERS)/American Thoracic Society (ATS) criteria (Chung et al., 2014). All 30 patients were treated with high-dose inhaled corticosteroids (ICSs) with long-acting β-adrenoceptor agonists (LABAs) and long-acting muscarinic receptor antagonists (LAMAs) and started biologic treatment with mepolizumab or omalizumab at enrolment, respecting the prescribing criteria (biological treatment—BT group). Phenotyping was carried out with additional standard investigations such as familiar, personal, and pathological medical history, lung function tests, blood tests (eosinophils count, total IgE), induced sputum, fractional exhaled nitric oxide (FeNO), and prick tests. The asthma control test (ACT) questionnaire was used to measure the degree of asthma control, and the asthma quality of life questionnaire (AQLQ) to evaluate the disease-specific health-related QoL. The first administration of the biologic agent is reported as T0. Subjects were revaluated at T1 (after 1 month), T3 (after 3 months), and T6 (after 6 months). During each examination, the following evaluations were performed: pulmonary function tests, measurement of FeNO, induced sputum collection, and processing, and assessment of daily life PA. For each test, the techniques are described in the following paragraphs.

We also enrolled a control group of 20 subjects (10 males and 10 females, mean age 62.7 ± 5.11 years) who were SA patients (diagnosed according to the ERS/ATS criteria) treated with traditional drug therapy (ICS/LABA/LAMA/oral corticosteroid—OC): traditional treatment—TT group.

Severe asthmatics who were enrolled in the study were assessed at least 4 weeks after an upper respiratory tract infection. Smokers and former smokers were excluded. No patient at enrolment had engaged in exercise training programs prior to the study.

Moreover, the experimental group was further subdivided into two groups, referring to the biologic therapy: omalizumab or mepolizumab. The omalizumab group was made up of 15 patients (10 females and 5 males, mean age 55.2 ± 10.4 years), while the mepolizumab group was made up of 15 patients (10 females and 5 males, mean age 55.2 ± 11.7 years).

**Figure 1:**
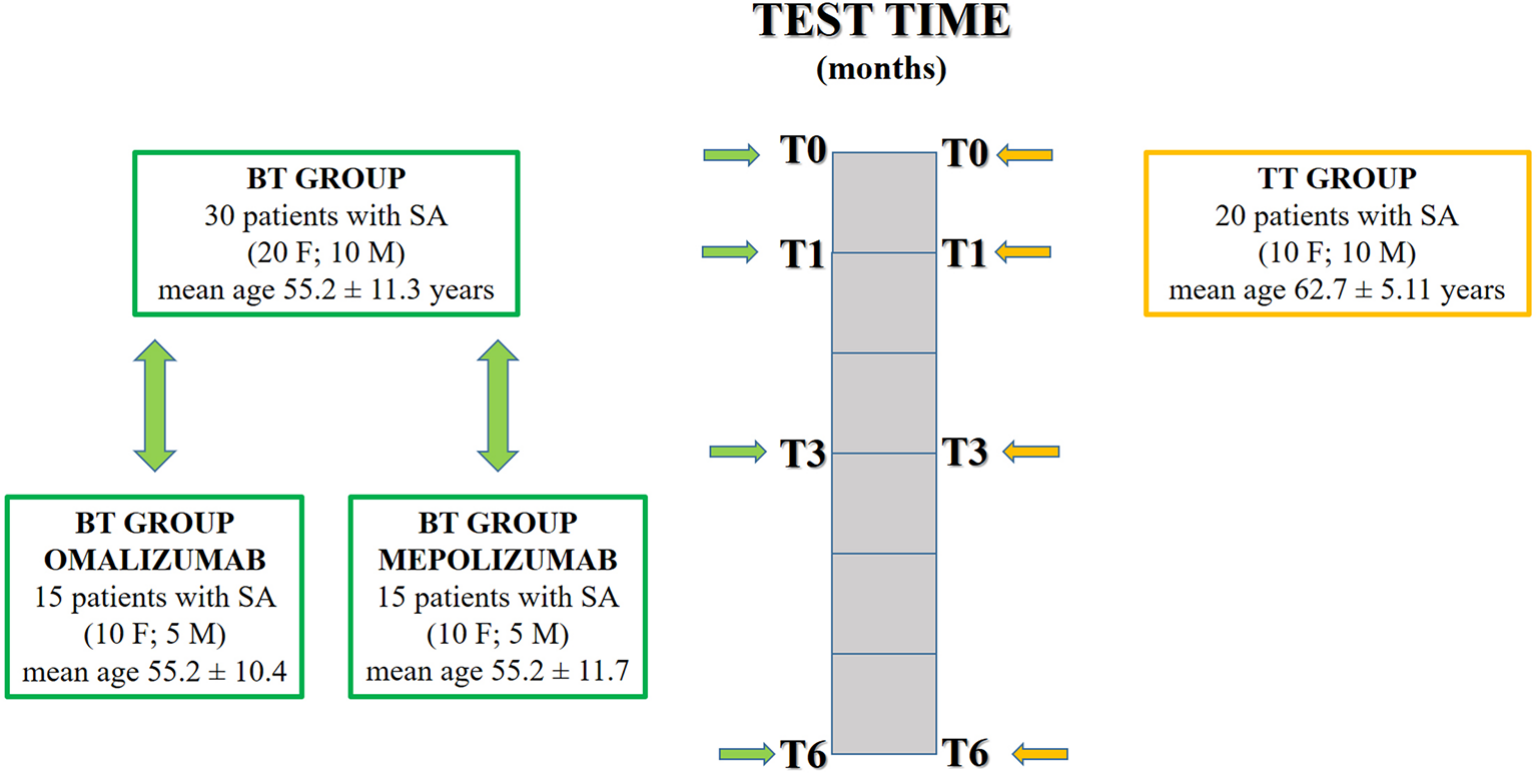
Two groups were enrolled: biological treatment group (BT group) and traditional treatment group (TT group). The BT group was further subdivided under drug criteria: omalizumab group and mepolizumab group. All groups were tested at different times: T0 (first administration of the biologic agent); T1 (after 1 month); T3 (after 3 months); T6 (after 6 months).

The study design is summarized in **Figure 1**.

This study was conducted in accordance with the amended Declaration of Helsinki. Institutional Ethics Committee of the University of Foggia approved the protocol (institutional review board approval N° 17/CE/2014), and written informed consent was obtained from all participants.

### Atopic Status

The skin prick test (SPT) was performed for a panel of inhalant allergens as previously described for common aeroallergens (Lofarma, Milan, Italy).

### Pulmonary Function Tests

To evaluate pulmonary function two different tests were performed using a spirometer (Sensormedics, Yorba Linda, CA, USA): forced expiratory volume in 1 s (FEV1) and forced vital capacity (FVC). Three maneuvers were carried out, recording the best value as a percentage of the predicted normal value. After baseline evaluation, spirometry was repeated 15 min after the subjects had inhaled 400 mg of salbutamol. Reversibility of airway obstruction was expressed in terms of the percent changes from baseline of the FEV_1_.

### Measurement of Fractional Exhaled Nitric Oxide

The Medisoft FeNO device (HYPAIR, Medisoft, Dinant, Belgium), which is semi-portable for repeatable multi-flow measurements of exhaled NO with off-line measurement, was used. Exhaled NO (FeNO) was measured with a constant expiratory flow of 50 ml. Repeated exhalations were performed until three plateaus, within 5% of inter-observation difference, were reached.

### Induced Sputum Collection and Processing

Sputum was induced through inhalation of hypertonic saline solution (4.5%) with an ultrasonic nebulizer (DeVilbiss 65; DeVilbiss Corporation, Somerset, PA, USA) and analyzed after selection of mucus plugs. All subjects were able to produce adequate sputum samples (defined as containing at least 500 non-squamous cells). The sputum (spontaneous or induced) was used for cytological analysis.

### Assessment of Daily Life Physical Activity

Daily life PA was recorded with a PA monitor (SenseWear^®^ Pro armband and SenseWear software version 8.0; BodyMedia Inc., Pittsburgh, PA, USA). All subjects were invited to wear the device continuously, for 5 consecutive days (3 week days and 2 weekend days), except while showering or bathing.

The armband was worn on the upper right arm. This multiaxial device has been validated in diverse populations, including patients with chronic diseases (St-Onge et al., 2007; Bahmer et al., 2017).

Four parameters were evaluated: the average number of steps per day (SPD), the average time (min/day) spent on activities with an estimated energy expenditure (EE) of ≥3 metabolic equivalents (METs), the average EE spent on activities requiring ≥3 METs (kcal/day), and the average total daily EE (kcal/day). METs reflect the energy cost of PA as a multiple of the patient’s resting metabolic rate. 20 EE ≥ 3.0 METs is considered to be at least moderate activity (Messina et al., 2017a; Zhu et al., 2018).

### Statistical Analysis

Statistical analysis was performed using SAS statistical software 9.3 (SAS Institute Inc., Cary, NC, USA) and GraphPad Prism 5 (GraphPad Software Inc., La Jolla, CA, USA). Normal distribution of the data was checked graphically and using the Shapiro-Wilk test. Continuous variables are expressed as mean ± standard deviation (SD) or median and range (min–max). Categorical variables are presented as frequencies and percentages. Categorical parameters were compared by the one-way ANOVA and *post-hoc* pair wise comparisons were performed with Tukey’s honestly significant difference (the TukeyHSD function in R). Correlations were assessed by calculating Pearson’s correlation coefficients. P < 0.05 was considered a statistically significant difference.

## Results

The baseline characteristics of the patients at T0 are summarized in **Table 1**.

**Table 1:** Population baseline characteristics at T0.

Characteristics	TT 20	BT 30	BT mepolizumab 15	BT omalizumab 15
Age, years	62.7 ± 5.11	55.2 ± 11.3	55.2 ± 11.7	55.2 ± 10.4
BMI, kg/m^2^	35.8 ± 6.27	29.32 ± 6.63	30.06 ± 5.75	28.57 ± 7.12
FEV1, % predicted	76.83 ± 9.58	77.62 ± 10.42	82.07 ± 8.77	72.6 ± 15.23
FVC, % predicted	101.13 ± 10.93	102.26 ± 7.37	108.25 ± 13.26	96.8 ± 3.9
FEV1/FVC, %	62.13 ± 8.43	64.19 ± 8.63	66.2 ± 6.36	62.12 ± 11.43
Age at diagnosis, years	44.3 ± 11.34	37.13 ± 14.07	35.46 ± 16.23	38.8 ± 11.85
ACT score, n	16.3 ± 6.53	14.4 ± 5.76	14.61 ± 5.18	13.77 ± 6.13
AQLQ score, n	4.53 ± 1.56	4.91 ± 1.81	4.84 ± 2.12	4.61 ± 0.9
FeNO50, ppb	29.26 ± 6.37	28.57 ± 5.29	23.49 ± 5.29	23.2 ± 3.77
Eosinophils in blood, %	2.83 ± 0.06*	5.08 ± 1.02*	4.83 ± 0.08	3.79 ± 0.06
Eosinophils in sputum, %	12.4 ± 18.68*	20.4 ± 24.87*	16.6 ± 24.76	14.4 ± 21.01
Nasal poliposis, %	12.7%	13.8%	14.6%	11.1%
GERD, %	19.05%	18.05%	25%	11.1%
ICS (high dose)/LABA/LAMA, (%)	93.8%	86.6%	—–	—–
ICS (low/moderate dose)/LABA, (%)	6.2%	13.4%	—–	—–

*p < 0.05. BMI, body mass index; FEV_1_, forced expiratory volume in 1 s; FVC, forced vital capacity; ACT, asthma control test; AQLQ, asthma quality of life questionnaire; FeNO, fractional exhaled nitric oxide; GERD, gastroesophageal reflux disease; ICS, inhaled corticosteroids; LABA, long acting beta2 agonist; LAMA, long acting muscarinic antagonist. Data are presented as the mean ± SD and %.

**Table 2** summarizes all the parameters analyzed in the two groups during the observational period (6 months).

**Table 2:** Physical activity, asthma control test (ACT), and asthma quality of life questionnaire (AQLQ) scores at T0, T1, T3, and T6 in biologic therapy (BT) and traditional treatment (TT) groups.

Parameters	TT				BT			
Experimental times	T0	T1	T3	T6	T0	T1	T3	T6
SPD, n	3,833.5 ± 639	4,043 ± 663.7	4,273 ± 619.1	4,528 ± 974.6	3,806 ± 421.6	4,438.8 ± 494.9	5,242 ± 653.7	6,545.1 ± 844.1
Total EE, kcal/day	1,324 ± 287.04	1,380.8 ± 292.5	1,476.8 ± 216.1	1,532.5 ± 216.1	1,318 ± 159.53	1,593.5 ± 184.36	1,819.2 ± 231.2	2,128.3 ± 435.9
ACT score, n	16.4 ± 6.34	15.7 ± 4.18	16.1 ± 8.12	18.4 ± 2.34	14.3 ± 5.84	18.1 ± 2.42	19.5 ± 4.28	24.3 ± 0.15
AQLQ score, n	4.55 ± 1.65	4.17 ± 1.85	4.39 ± 1.17	4.87 ± 1.03	4.86 ± 1.94	4.97 ± 1.58	5.16 ± 1.14	5.78 ± 1.25

SPD, number of steps per day; EE, estimated energy expenditure; ACT, asthma control test; AQLQ, asthma quality of life questionnaire. Data are presented as the mean ± SD.

**Figure 2:**
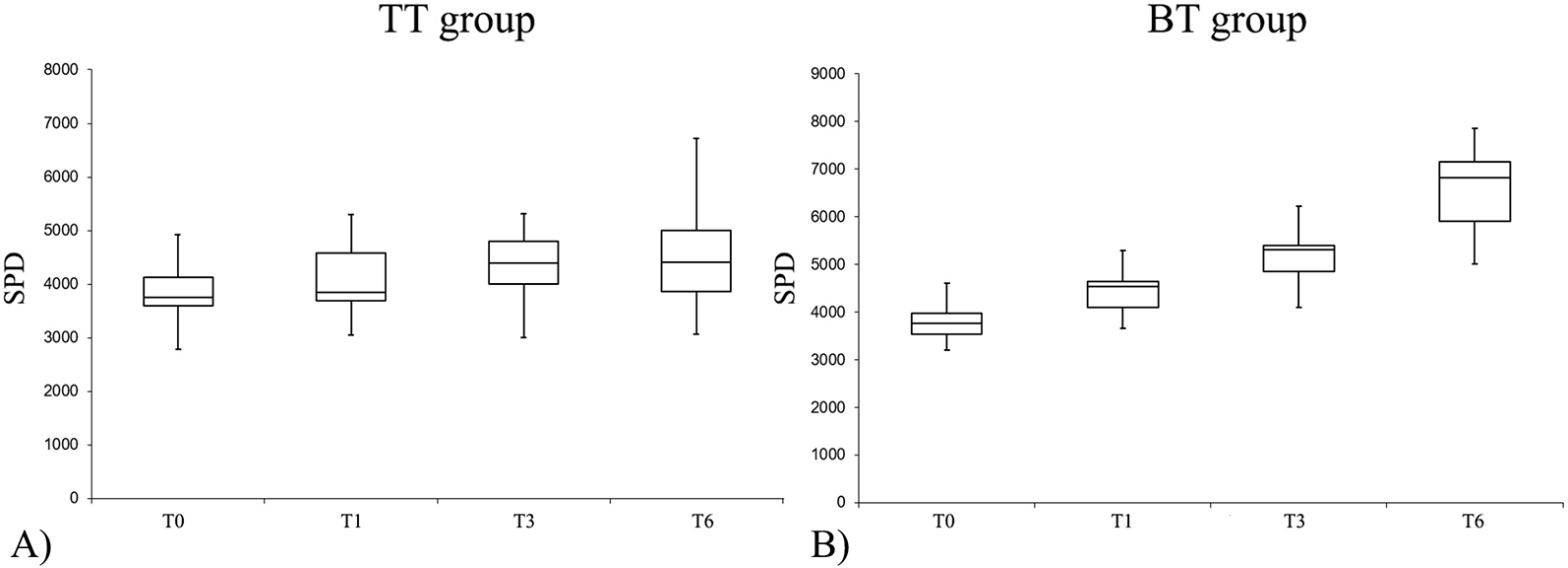
Number of SPD (steps per day) variation at different experimental times in the TT (traditional therapy) group **(1A)** and in the BT (biological therapy) group **(1B)**.

The PA levels were significantly improved at T6 compared to T0 (**Figure 2**) during follow-up, considering the intragroup variation, both in the BT group and in the TT group. Considering the intragroup variation, it is very interesting to note that biologic therapy improved PA levels compared to the effects of traditional therapy; indeed, while at T0 there were no significant differences in the SPD values between the two groups (T0, p = 0.85), the differences become statistically significant at T1, T3, and T6 (T1, p = 0.019; T3, p = 3.48x10^−6^; T6, p = 4.78 x 10^−10^).

As expected, the same differences were reported analyzing the data from the EE: considering the intragroup variation, in both groups the PA levels improved comparing the data at T0 with T6 (p < 0.5). To evaluate the effects of the biological drug compared to traditional treatment, the differences between T0 and T6 were analyzed: at the start point, there were no statistical differences between the two groups, while it became significant during follow-up.

The main data of the biologic drugs used in the treatment are reported in **Table 3** (omalizumab *vs.* mepolizumab).

**Table 3:** Physical activity at T0, T1, T3, and T6 in biologic therapy (BT) omalizumab and BT mepolizumab groups.

Parameters	BT omalizumab				BT mepolizumab			
Experimental times	T0	T1	T3	T6	T0	T1	T3	T6
SPD, n	3,818.1 ± 454.84	4,525 ± 496.5	5,106 ± 616.7	6,508.53 ± 930.9	3,794.13 ± 401.3	4,352.13 ± 494.6	5,379.6 ± 681.7	6,581.66 ± 778.8
Total EE, kcal/day	1,325.2 ± 161.4	1,604± 177	1,807.4 ± 219.5	2,121 ± 394.9	1,310.9 ± 162.9	1,583 ± 197	1,831 ± 249.6	2,132.6 ± 487.4

SPD, number of steps per day; EE, estimated energy expenditure. Data are presented as the mean ± SD.

No significant differences in PA levels were found between the two BT groups (mepolizumab *vs.* omalizumab), while both drugs produced a positive effect on PA levels during follow-up.

**Figure 3:**
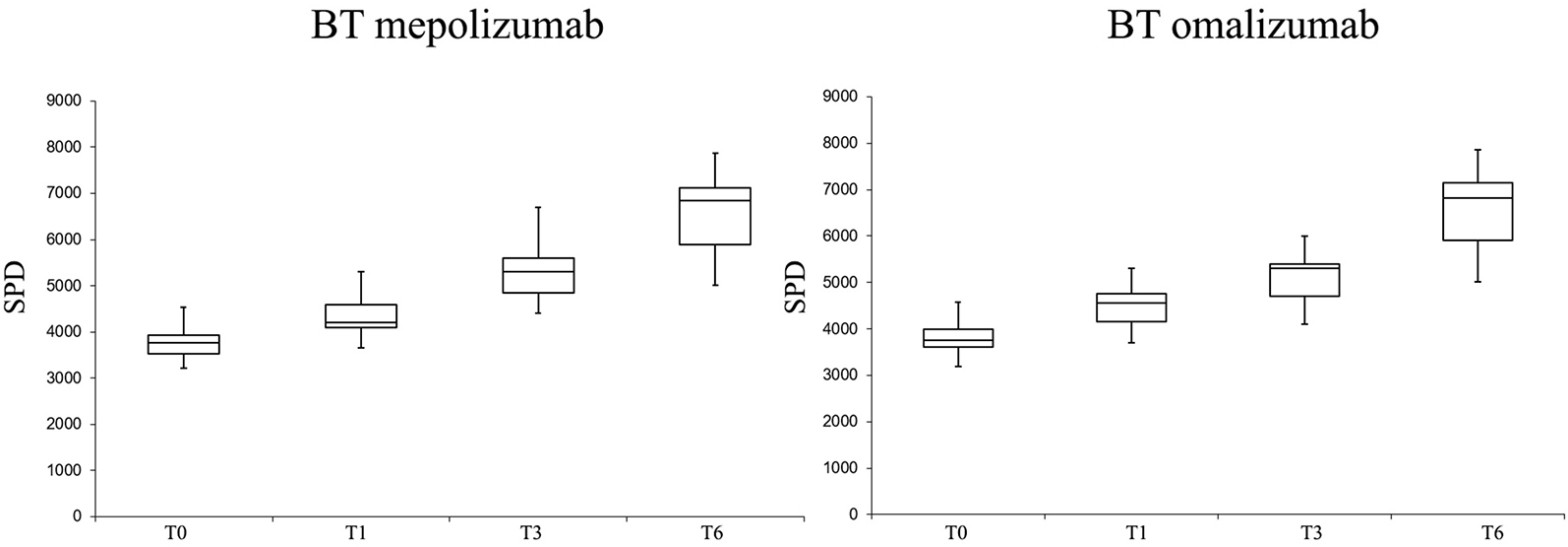
Number of steps per day (SPD) variations at different follow-ups in the biologic therapy (BT) omalizumab group and in the BT mepolizumab group.

**Figure 4:**
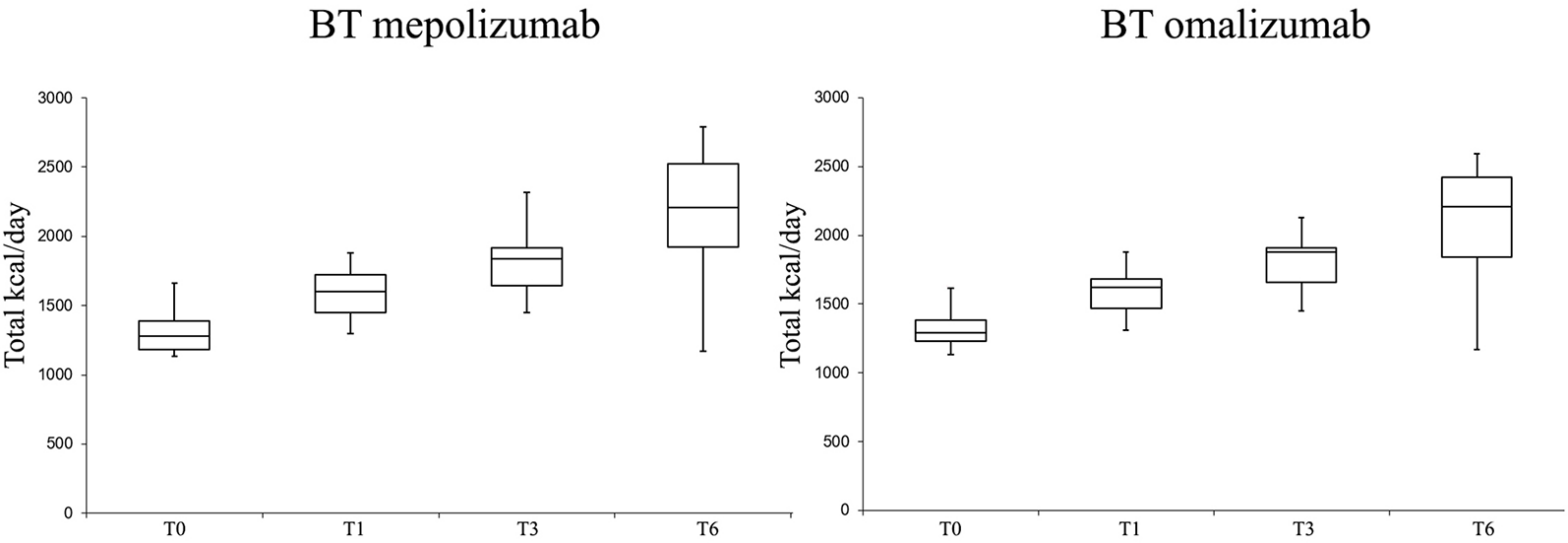
Total EE (estimated energy expenditure) variation at different follow-ups in the biologic therapy (BT) omalizumab group and in the BT mepolizumab group.

PA levels in terms of SPD and EE were significantly improved in both groups with the biologic therapies (T6 *vs.* T0) (**Figures 3** and **4**).

Furthermore, in the BT group the AQLQ score was significantly improved at T6 compared to T0 (4.86 ± 1.94 *versus* 5.78 ± 1.25, p < 0.05), while in the TT group, this difference was not significant (4.55 ± 1.65 *versus* 4.87 ± 1.03, p = 0.67). No significant differences were found between the two BT groups in terms of the AQLQ score.

After 6 months (T6), patients in the BT group showed a reduction in the number of exacerbations/year compared to T0 (3 ± 0.7 *vs.* 0.95 ± 0.75; p < 0.01), without significant differences between the two BT groups. On the contrary, patients of the TT group did not show differences at T6 in terms of number of exacerbations/year. No significant differences between groups were found at T6 in terms of FEV_1_.

Finally, we found a positive correlation between SPD and the AQLQ score (ρ = 0.44, p <0.05) but we did not find any correlations between SPD or total EE and BMI, FEV_1_, or ACT score.

## Discussion

The use of biologics (omalizumab, mepolizumab, reslizumab, benralizumab) in asthma patients has had promising preliminary results, even if other biologics have not shown a significant clinical response (Zhu et al., 2018). Mepolizumab has been shown, in previous studies, to reduce exacerbations and dependency on oral corticosteroids compared with placebo, improving the QoL of asthma patients. In the same way, omalizumab (the first Food and Drug Administration—FDA-approved biologic for the treatment of SA), in several clinical trials has shown clinical benefits in patients with severe allergic asthma (McCracken et al., 2016; Menzella et al., 2017). In a recent case report, Peterson and Coop described successful long-term use of omalizumab for exercise-induced anaphylaxis (EIA) in a subject who was refractory to traditional therapy (Peterson and Coop, 2017). Moreover, Oliveira et al. described the positive effects of omalizumab therapy, demonstrating a reduction of rescue medications and asthma exacerbations in the studied population; moreover, they reported an improved lung function in obese patients (Oliveira et al., 2019).

The preliminary results of this pilot study show, for the first time, that PA levels are significantly increased in adult patients with SA treated with biologics compared to those treated with traditional therapies.

Few studies have been published about PA levels in patients affected by SA, moreover, no previous studies described its changes during treatment. Cordova-Rivera et al. (2018b) demonstrated that compared to healthy controls, patients with SA, bronchiectasis, and chronic obstructive pulmonary disease (COPD) accumulated less SPD. Although SA is known to limit the PA of patients because of symptom perception and fear of worsening, to our knowledge this topic has never been explored in SA patients.

In our study, both SA groups showed an improvement in PA levels during drug treatment. A significant statistical difference was noted not only intergroup, by analyzing the base values (T0), with respect to T6, but also between the two groups. As reported in the *Results* section, the data about SPD demonstrated that PA was significantly higher in the BT group with respect to the TT group after 3 months and 6 months of therapy. Our results are in agreement with previous studies that analyzed the PA levels in patients with other respiratory diseases. Pitta et al. (2008) analyzed PA levels in patients with COPD, reporting that walking time in daily life did not improve significantly after 3 months of a multidisciplinary rehabilitation program, but only after 6 months. Thyregod et al. (2018), in their pilot study, reported that the median daily steps were significantly improved in patients with COPD responding to their rehabilitation program.

Based on biologic therapy, the differences in daily PA become very different comparing the two groups. Obviously, the EE levels were higher in the group treated with biologics, representing an important tool for helping patients with weight control. These findings suggest that the use of new biologic drugs in SA could greatly improve the QoL of these patients.

In a previous study, Chupp et al. reported that the use of mepolizumab in the treatment of asthma patients improved the health-related QoL, particularly in patients with severe eosinophilic asthma (Chupp et al., 2017). Canonica et al. demonstrated that the use of omalizumab in severe allergic asthma improves patient outcomes such as QoL and patients’ illness perception (Canonica et al., 2018). PA levels are strictly related to QoL: PA levels are improved in populations with a high QoL (Mazzeo et al., 2016; Monda et al., 2017b; Wu et al., 2017; Cordova-Rivera et al., 2018a; Sessa et al., 2018a). In our preliminary real-life study, for the first time, we demonstrated the improvement of the QoL of SA patients treated with biologics in terms of PA levels and, indirectly, in terms of the AQLQ score. Particularly, the present study highlights that prolonged therapy with biologics improved PA levels.

Evidence indicates that asthma patients limit exercise and healthy lifestyle activities to avoid respiratory symptoms. This self-imposed decrease in activity may predispose them to long-term general health risks. Mancuso et al. (2006) reported an interesting study based on interviews: although most patients knew the importance of PA, particularly linked to their health status, many did not usually exercise, reporting several barriers such as lack of motivation, time constraints, and extreme weather affecting asthma, thus leaving asthmatic subjects to become prisoners in their own homes and of their fears. Patients with more severe disease were more likely to believe that exercise was not good for asthma. Furthermore, the deprivation of those daily life aspects unavoidably has as a consequence on feelings of anxiety and depression.

In addition to social and personal reasons that support the usefulness of improving PA, there are also physiological aspects of PA that we need to remember. As well as those described in the literature, lower levels of sedentary time combined with higher levels of activity are associated with better asthma control, reducing oxidative stress, and improving QoL (Cordova-Rivera et al., 2018b).

On the one hand, in most children with asthma, exercise could be considered a trigger of asthma symptoms, inducing bronchospasm; it can also be a unique asthma phenotype (Chupp et al., 2017). On the other hand, it is well noted that low physical fitness in childhood is linked to the development of asthma in young adulthood (Koczulla et al., 2017; Aggarwal et al., 2018).

The present study demonstrates that biologic therapy could be very useful in increasing the PA of SA patients and this finding is in line with previous studies (Rasmussen et al., 2000; Garber et al., 2011; Van’t Hul et al., 2016).

In consideration of the important improvement that we demonstrated after BT, we suggest that PA could be considered another important tool in the evaluation of the response of SA treatment with biologics. We suggest that PA parameters should be measured in all subjects who start biologics and this could give important further information to evaluate the clinical response.

The main limitation of our study is the small number of subjects enrolled in both groups (BT *vs.* TT), even if the results suggest a positive relationship between the use of biologics, the QoL of the patients, and the PA of the same subjects. This limitation became very important analyzing the data about the two biologics (omalizumab *vs.* mepolizumab) used for experimentation. We can justify this with the low prevalence of SA and with the small number of subjects that met the prescription criteria. Moreover, the control group was made up of subjects that self-manage their asthma at home, even if they were checked at the same time as the subjects of the BT Group. In the light of these considerations, it is very important to consider this study as a pilot study to stimulate further studies. In this regard, we are planning a future study with a larger population to confirm our results.

## Conclusion

In conclusion, this pilot study proposes a positive relationship between biologic therapy and PA patterns, underlining the necessity to better explore this field. We believe that PA merits further consideration as an outcome of efficacy of biologic treatments, because improving PA means improving the health status of SA patients.

## Data Availability Statement

All datasets generated for this study are included in the article.

## Ethics Statement

The studies involving human participants were reviewed and the protocol was approved by the Institutional Ethics Committee of the University of Foggia (institutional review board approval N° 17/CE/2014). The patients/participants provided their written informed consent to participate in this study.

## Author Contributions

GEC, FS, GS, DL and GM confirmed that the study objectives and procedures are honestly disclosed; moreover, GEC, FS, MF and GM reviewed study execution data and confirmed that procedures were followed to an extent that convinces all authors that the results are valid and generalizable to a population similar to that enrolled in this study. GEC, FS, GS, AV, AT, GC and GM also contributed to drafting the manuscript for content, study content or design and analysis, and interpretation of data. DL, MV, AT, MF, GC and GM contributed to statistical analysis. FS, GS, MF, OR, GC, AT, DL and MV contributed to revising the submitted article critically and substantially for important intellectual content.

## Conflict of Interest

The authors declare that the research was conducted in the absence of any commercial or financial relationships that could be construed as a potential conflict of interest.
